# The Impact of Excluding Nonrandomized Studies From Systematic Reviews in Rare Diseases: “The Example of Meta-Analyses Evaluating the Efficacy and Safety of Enzyme Replacement Therapy in Patients With Mucopolysaccharidosis”

**DOI:** 10.3389/fmolb.2021.690615

**Published:** 2021-06-22

**Authors:** Miguel Sampayo-Cordero, Bernat Miguel-Huguet, Andrea Malfettone, José Manuel Pérez-García, Antonio Llombart-Cussac, Javier Cortés, Almudena Pardo, Jordi Pérez-López

**Affiliations:** ^1^ Medica Scientia Innovation Research (MedSIR), Barcelona, Spain; ^2^ Colorectal Unit, Department of Surgery, Hospital de Bellvitge, Barcelona, Spain; ^3^ IOB Institute of Oncology, Quiron Salud Group, Madrid, Spain; ^4^ Hospital Arnau de Vilanova, Universidad Católica de Valencia San Vicente Mártir, Valencia, Spain; ^5^ Vall d´Hebron Institute of Oncology (VHIO), Barcelona, Spain; ^6^ Albiotech Consultores y Redacción Científica S.L., Madrid, Spain; ^7^ Department of Internal Medicine, Hospital Vall d’Hebron, Barcelona, Spain

**Keywords:** systematic review, mucopolysaccharidosis, enzyme replacement therapy, rare disease, nonrandomized study, meta-analysis, case reports

## Abstract

Nonrandomized studies are usually excluded from systematic reviews. This could lead to loss of a considerable amount of information on rare diseases. In this article, we explore the impact of excluding nonrandomized studies on the generalizability of meta-analyses results on mucopolysaccharidosis (MPS) disease. A comprehensive search of systematic reviews on MPS patients up to May 2020 was carried out (CRD42020191217). The primary endpoint was the rate of patients excluded from systematic reviews if only randomized studies were considered. Secondary outcomes included the differences in patient and study characteristics between randomized and nonrandomized studies, the methods used to combine data from studies with different designs, and the number of patients excluded from systematic reviews if case reports were not considered. More than 50% of the patients analyzed have been recruited in nonrandomized studies. Patient characteristics, duration of follow-up, and the clinical outcomes evaluated differ between the randomized and nonrandomized studies. There are feasible strategies to combine the data from different randomized and nonrandomized designs. The analyses suggest the relevance of including case reports in the systematic reviews, since the smaller the number of patients in the reference population, the larger the selection bias associated to excluding case reports. Our results recommend including nonrandomized studies in the systematic reviews of MPS to increase the representativeness of the results and to avoid a selection bias. The recommendations obtained from this study should be considered when conducting systematic reviews on rare diseases.

## Introduction

The randomized clinical trials provide the strongest evidence regarding the efficacy of new therapeutic interventions (Higgins et al., 2019). Thus, they must be eligible for systematic reviews. On the contrary, nonrandomized designs, in addition to different design features, are variable in their susceptibility to bias (Reeves et al., 2017). Empirical evidence suggests that the observational studies are considered less rigorous, without a preplanned statistical analysis plan, with a higher risk of selection bias, and more affected by confounding than randomized clinical trials (Higgins et al., 2019; Morgan et al., 2019). Furthermore, the systematic review guidelines recommend that both randomized and nonrandomized studies with different design features should be analyzed separately (Higgins et al., 2019). Accordingly, nonrandomized studies and case reports are excluded from systematic reviews and only considered when there are no other design alternatives (Catalá-López et al., 2017; Pérez-López et al., 2017; Sampayo-Cordero et al., 2018; Sampayo-Cordero et al., 2019).

In the field of rare diseases, and specifically in mucopolysaccharidosis (MPS), the low prevalence of the disease makes the possibility of performing randomized clinical trials extremely difficult. Additionally, clinical trials usually include a relatively more homogeneous population according to predefined characteristics than clinical practice. Thus, the phenotypic and genotypic heterogeneity increases the difficulty to generalize the efficacy results from a clinical trial (Kahan et al., 2015; Frieden, 2017; Hong et al., 2018). Previous systematic reviews have stated that observational studies properly conducted could achieve evidence equivalent to that of randomized clinical trials (Concato et al., 2000; Sampayo-Cordero et al., 2018; Sampayo-Cordero et al., 2019). It is increasingly recognized that subject selection criteria and monitoring conditions of clinical trials usually differ from clinical practice, while prospective studies and case reports are nearer to standard clinical practice conditions (Kahan et al., 2015; Hong et al., 2018; on behalf of the ACMG Professional Practice and Guidelines Committee et al., 2020). Therefore, the exclusion of patients from nonrandomized studies and case reports could reduce the generalization of the meta-analyses results and introduce a selection bias in the study. Moreover, different methods have been proposed to combine information provided by clinical trials, clinical studies, and case reports (Bradley et al., 2017; Dornelles et al., 2017; Pérez-López et al., 2017, Pérez-López et al., 2018).

Previous systematic reviews on patients with MPS have evaluated the efficacy and safety of enzyme replacement therapies (ERTs). However, the selection strategies among different revisions and meta-analyses are quite heterogeneous. Some studies included only randomized trials and others allowed for the selection of different types of nonrandomized designs (Schrover et al., 2017; Gomes et al., 2019; Jameson et al., 2019).

Our proposal is to carry out a systematic review of meta-analyses that includes randomized or nonrandomized studies in MPS that evaluate the efficacy or safety of ERTs. The aim of the present study is to explore the impact of excluding nonrandomized studies on the generalizability of the meta-analysis results and the probability of selection bias. Additionally, we report the methods used to combine data from different study designs.

## Materials and Methods

### Data Sources and Searches

A comprehensive search of systematic reviews containing clinical information of MPS patients up to May 2020 was carried out on MEDLINE, the Cochrane Library (Cochrane Database of Systematic Reviews and protocols), and the International Prospective Register of Systematic Reviews (PROSPERO) databases as well as on the Latin American and Caribbean Literature on Health Sciences (LILACS). The search strategy retrieved citations from the MEDLINE database containing the “All fields” headings: “mucopolysaccharidosis” and “systematic review”. In the Cochrane Library, the strategy retrieved citations containing the “complete text” headings: “mucopolysaccharidosis”. The search strategy for the PROSPERO database retrieved citations containing the heading: “mucopolysaccharidosis”. Finally, the search strategy for the LILACS database retrieved citations containing the “Title, Summary, Issue” headings: “mucopolysaccharidosis” and “systematic review”. There was no restriction of dose, treatment duration, administration (*via* intravenous or intrathecal), type of study design, or language. All published studies up to May 20, 2020, were included in the search.

### Study Selection

The original articles of the systematic reviews selected by an electronic search were obtained and reviewed. We selected those systematic reviews meeting the following selection criteria:

#### Inclusion Criteria

a) Systematic reviews of nonrandomized and/or randomized studies conducted in patients with MPS.
b) Systematic reviews based on the assessment of ERT efficacy and safety.

#### Exclusion Criteria

a) Nonsystematic reviews.
b) Systematic reviews evaluating pharmacoeconomic endpoints, preclinical evaluations, or incidence of MPS.
c) Systematic reviews evaluating only hematopoietic stem cell transplantation.

### Quality Assessment

The study was prospectively designed to describe the methods used in MPS systematic reviews. The current meta-analysis is reported in accordance with the preferred reporting items for systematic reviews and meta-analysis (PRISMA) and meta-analyses and systematic reviews of observational studies (MOOSE) guidelines (Stroup et al., 2000; Liberati et al., 2009; Page and Moher, 2017). The protocol was published in the PROSPERO database (CRD42020191217). Two investigators entered findings into a database, independently reviewed citations/abstracts from the database and hand-searched and selected full relevant articles and documents for data extraction using the abovementioned preset criteria. Discrepancies were resolved through discussion or input from a third reviewer. The contributions of all authors are described in the author’s contributions section.

### Primary Outcome

The primary outcome is the number of patients from nonrandomized studies (clinical studies and case reports) included in the MPS reviews among all patients included (i.e., patients from both randomized and nonrandomized studies). The number of patients from nonrandomized studies was calculated for each clinical type of MPS. When there was more than one systematic review for an MPS type, we selected the systematic review with more patients included. We have not summed all the patients from each systematic review because we would have counted some patients more than once.

This measure was calculated to estimate the rate of patients excluded from the meta-analyses and qualitative synthesis where only randomized studies have been considered. Statistical guidance has stated that the more the missing values, the less the grade of evidence obtained (Little et al., 2012; Madley-Dowd et al., 2019). Patients who were reported in more than one nonrandomized study were counted once (Bradley et al., 2017).

However, in some randomized clinical trials, the patient follow-up was extended after completing the study and the placebo arm started to receive ERT, thus switching the study design to a prospective nonrandomized design for the analysis of long-term outcomes. As the study design and the evaluations performed in the randomized and nonrandomized stages of these trials were different, their patients have been considered twice (Wraith et al., 2004; Muenzer et al., 2006; Muenzer et al., 2011; Clarke et al., 2009; STRIVE; Investigators et al., 2014; Hendriksz et al., 2016).

### Secondary Outcomes

1) We qualitatively described the differences in patients selected and study conduct between randomized and nonrandomized studies included in a systematic review of the same MPS type. We evaluated these differences at four levels: treatment schedule, patients’ characteristics, period of follow-up, and outcomes. The recommendations for the management of missing values in clinical trials suggest that the proportion of missing values is not as relevant as the reason and pattern. Thus, it was important to explore if these missing values were at random, or if there were differences in baseline characteristics between patients included and not included in the analyses (Little et al., 2012; Madley-Dowd et al., 2019).
2) We described the methods used to combine data from different study designs.
3) We estimated the number of patients who were included in a systematic review excluding case reports among all patients included in randomized, nonrandomized, and case reports studies. This measure calculates the number of patients excluded from the meta-analyses and qualitative synthesis if case reports were not considered. As stated earlier, patients included in both randomized trials and prospective study extensions were considered twice (Wraith et al., 2004; Muenzer et al., 2006; Muenzer et al., 2011; Clarke et al., 2009; STRIVE; Investigators et al., 2014; Hendriksz et al., 2016).

### Statistical Methods

The primary variable is the rate of patients excluded from the meta-analyses and qualitative synthesis if only randomized studies were to be considered. The primary variable is reported as the percentage of patients for each MPS. Statistical guidance in single clinical trials has stated that bias is likely in analyses with more than 10% of missing data and that if more than 40% of data are missing in important variables, results should only be considered as hypothesis-generating (Little et al., 2012; Madley-Dowd et al., 2019). Thus, we tested the null hypotheses that the rate of patients excluded when nonrandomized studies are not considered is less than or equal to 10% (likely bias) and that is less than or equal to 40% (evidence degraded to exploratory). Both null hypotheses have been tested with a fixed sequence approach. First, we compared the observed rate and the exclusion rate with a 10% null hypothesis and continued with the next analysis (40% null hypothesis), only if the previous assessment met the requirements for statistical significance. We based our analysis on a one-sided binomial test (Sampayo-Cordero et al., 2020b). This primary objective was analyzed with a nominal alpha level of 0.025 one-sided (equivalent to 0.05 two-sided). Multiplicity issues derived from analyzing the rate of excluded patients in each MPS type were corrected based on the Bonferroni method. We multiplied the *p*-values by the number of MPS analyzed (U.S. Department of Health and Human Services Food and Drug Administration et al., 2017). In addition, we also reported the 95% confidence interval for the rate of patients excluded.

We summarized in each systematic review selected, the differences in patients selected and study conduct between randomized and nonrandomized studies, describing them in a narrative format for each MPS type. Additionally, the methods used to combine data from different study designs were reported in the same format.

The rate of patients excluded from the meta-analyses and qualitative synthesis, if case reports were not considered, was also analyzed in accordance with the primary endpoint methods. However, multiplicity adjustment was not performed.

## Results

### Data Search Results

**FIGURE 1:**
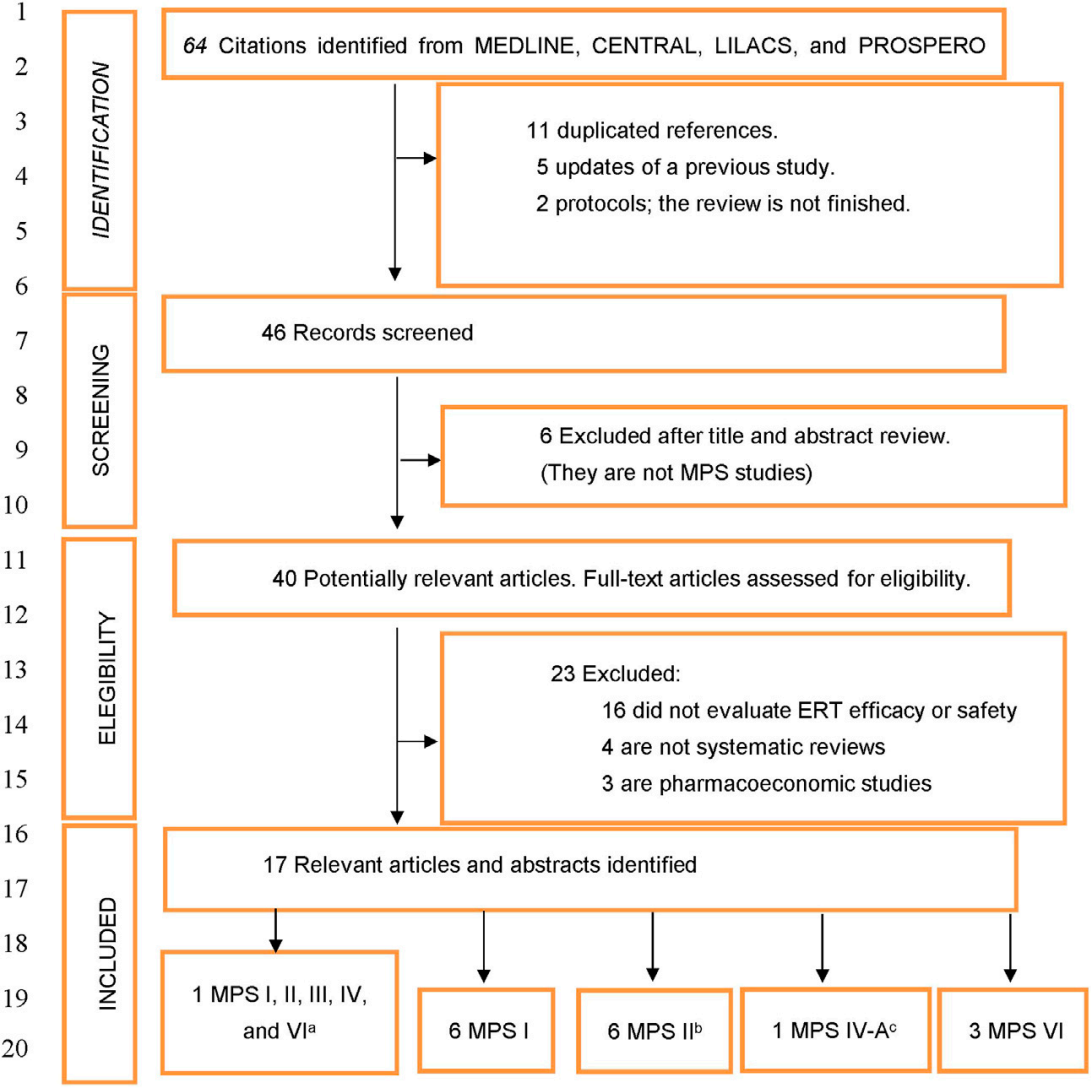
Flow diagram of systematic reviews that evaluated the efficacy and safety of enzyme replacement therapies in mucopolysaccharidosis. MPS, Mucopolysaccharidosis; ERT, Enzyme replacement therapy. ^a^ One systematic review included all types of MPS patients to evaluate the pretreatment and posttreatment prevalence and severity (Pal et al., 2015). This systematic review did not include outcomes of ERT for MPSIII and MPS IV. ^b^ We included a congress communication evaluating enzyme replacement therapy safety in MPS II (Almeida et al., 2018). ^c^ This systematic review only includes patients with Morquio A syndrome.

Database searches through May 20, 2020, identified 64 citations and 46 unique abstracts. Among the 46 studies identified, six were excluded after the abstract revision because they were not MPS studies. A total of 23 out of 40 communications with a full text review were excluded because they did not evaluate ERT efficacy or safety (*N* = 16), were not systematic reviews (*N* = 4), or were pharmacoeconomic studies (*N* = 3). Thus, 17 reviews which met the inclusion criteria and evaluated ERT efficacy or safety in patients with MPS (*N* = 6 in type I MPS, *N* = 6 in type II MPS, *N* = 1 in type IV-A MPS, *N* = 3 in type VI MPS, and *N* = 1 including patients with type I to type VI MPS) were included in the present study. The references of all abstracts screened and the reasons for exclusion are reported in Figure 1; Supplementary Material Table S1. The publication years ranged between 2007 and 2019 (see Table 1).

**TABLE 1:** Rate of patients excluded from systematic review when nonrandomized studies are not included.

Type of MPS	Study	Patients included in systematic review			Rate of patients excluded	Multiplicity adjusted one-sided *p*-value	
		Randomized trial	Nonrandomized trial	Total	95% Confidence interval	10% null hypothesis	40% null hypothesis
Types I, II, III, IV, and VI	Pal et al. (2015)	52	281^a^	333	84.4 (80–88.1%)	< 0.0001	< 0.0001
Type I	All selected	max = 45	max = 216	261	82.8 (77.6–87.1%)	< 0.0001	< 0.0001
	El Dib and Pastores, (2007)	45	0	45			
	Dornelles et al. (2017)	45	142^a^	187			
	Pérez-López et al.	12	45^a^ ^,^ ^b^	57			
	Sampayo-Cordero et al. (2018a)	0	11	11			
	Jameson et al. (2019)	45	0	45			
	Kuiper et al. (2019)	0	216	216			
Type II	All selected	max = 108	max = 1216	1,324	91.8 (90.2–93.3%)	<0.0001	<0.0001
	Alegra et al. (2013)	108	110 ^a^	218			
	da Silva et al. (2016)	96	0	96			
	Almeida et al. (2018)	108	1,216 ^a^	1,324			
	Pérez-López et al.	3	27 ^a^ ^,^ ^c^	30			
	Bradley et al. (2017)	108	820 ^a^	928			
	Sampayo-Cordero et al. (2019)	0	44	44			
Type IV-A	Schrover et al. (2017)	176	173 ^a^	349	49.6 (44.2–54.9%)	<0.0001	0.0003
Type VI	All selected	max = 46	max = 362	408	88.7 (85.2–91.6%)	<0.0001	<0.0001
	El Dib (2009)	46	0	46			
	Brunelli et al. (2016)	39	0	39			
	Gomes et al. (2019)	0	362	362			

Max: The maximum number of patients included in a systematic review for this MPS type. We selected this method of aggregation over sum or mean, because different systematic reviews compare the same patients and the same evaluations.

a The studies included a randomized phase III trial and its follow-up extension. The placebo arm started to receive enzyme replacement therapy and thus, the study switched to a prospective nonrandomized design for the long-term outcomes. As the study designs and evaluations performed were different, the information provided for the same patient was different. So, these patients have been considered twice; as participants of the randomized studies and as participants of the nonrandomized studies (21–26).

b According to the study objectives, only adult patients were considered (≥18 years).

c According to the study objectives, only adult patients were considered (≥16 years).

### Primary Outcome

The rate of patients missing from systematic reviews when nonrandomized studies were excluded was significantly higher (*p* < 0.001) than 40% in all MPS types (Table 1). We observed that at least 50% of MPS patients had been recruited in nonrandomized designs. In accordance with the rate of missing boundaries proposed, the results of MPS systematic reviews excluding nonrandomized trials should not be considered confirmatory but only hypothesis generating (see Table 1) (Little et al., 2012; Madley-Dowd et al., 2019).

### Secondary Outcomes

#### Differences in Patients Included and Trials Conduct Between Randomized and Nonrandomized Studies Selected in Systematic Reviews

The qualitative analysis of patients included in randomized and nonrandomized studies suggested relevant differences in dose treatment schedule, inclusion criteria, extension of follow-up, and outcomes evaluated between the two types of studies. Therefore, there were some types of patients (with less frequent phenotypes, the youngest (<6 years) and oldest patients (>50 years), and special populations (pregnant women, infants, and suitable patients for HSCT and intrathecal therapies)), dose schedules, and clinically relevant outcomes (such as mortality, long-term efficacy, and extended safety profile) that were excluded from randomized trials. Even in the studies evaluating phase III extended follow-up with the same patients included in the randomized and prospective designs (placebo arm received ERT in an extended follow-up), the nonrandomized study provided results about long-term efficacy and safety, and randomized trials excluded these relevant outcomes (see Table 2).

**TABLE 2:** Differences in patients included and trial conduct between randomized and nonrandomized studies selected in systematic reviews.

Type	Parameter evaluated	Main differences in patients selected and study conduct between	
		Randomized trials	Nonrandomized studies
Types I, II, III, IV, and VI	Dose and schedule	For laronidase: weekly intravenous infusions of 0.58 mg/kg	Additional doses and therapies were considered (HSCT). However, the doses and therapies evaluated at clinical trials are the most common
		For idursulfase: 0.5 mg/kg/week or every other week	
	MPS phenotype	The study only includes randomized trials of MPS I and II patients. Clinical trials only include Huler–Scheie and Scheie phenotypes for MPS I and attenuated patients for MPS II.	Patients with MPS type III, IV, and VI were also evaluated
			They include a broad range of phenotypes for MPS I and II.
	Follow-up time	Follow-up restricted to a maximum of 26 weeks in MPS I and 12 months in MPS II clinical trials. There was no evaluation of long-term outcomes	The follow-up was extended in some studies from 2 to 6 years
	Outcomes	Role of ERT in the improvement of obstructive sleep apnea	They also considered other outcomes: prevalence and severity of obstructive sleep apnea and the role of HSCT.
Type I	Dose and schedule	Weekly intravenous infusions of 0.58 mg/kg	Additional doses were weekly intravenous infusions of 0.54, 1.16, 1.2, and 1.8 mg/kg. The intrathecal administration was also evaluated. However, the most common schedule was weekly intravenous infusions of 0.58 mg/kg
	MPS phenotype	The study only includes 1 patient with hurler phenotype (later it was reclassified at Hurler–Scheie phenotype) and 7 patients with Scheie phenotype. The most patients included had Hurler–Scheie phenotype (*n* = 37)	There are more patients of Hurler and Scheie phenotypes
	Follow-up time	Follow-up restricted to 26 weeks. No evaluation of long-term outcomes	The follow-up was extended in most of studies to 52 weeks and more (288 weeks). Evaluation of long-term outcomes
	Outcomes	6-MWT, urinary glycosaminoglycan levels, liver and spleen volume, forced vital capacity, articular flexion, level of antibodies to laronidase, nocturnal hypoventilation/sleep apnea, quality of live, adverse events, and tolerability	They also considered other outcomes: mortality, other anatomical and organ characteristics, visual acuity, intraocular pressure, neurological symptoms, cognitive evaluation, clinical development, post-surgery complications, cardiac symptoms, dermatological outcomes, bone mineral density, other respiratory endpoints, other cardiac endpoints, tracheal evaluation, sleep apnea, growth evolution, earing function, and clinical endpoints in a pregnant woman and her infant
Type II	Dose and schedule	0.5 mg/kg/week or every other week of idursulfase	Additional doses were weekly intravenous infusions of 0.3, 0.4, and 0.1 mg/kg idursulfase. Idursulfase beta was also considered (0.5 and 1 mg/kg). However, the most common schedules were 0.5 mg/kg/week or every other of idursulfase
	Muchopolysaccharidosis phenotype	Studies only include patients with attenuated phenotype	Studies include more patients with severe phenotype
	Follow-up time	Follow-up restricted to 12 months. No evaluation of long-term outcomes	The follow-up was extended in most of studies to 24 months and more (288 months). Evaluation of long-term outcomes
	Outcomes	6-MWT, liver and spleen size, urinary glycosaminoglycan levels, cardiac function, pulmonary function, sleep disorders, level of antibodies to idursulfase, adverse events, and tolerability	They also considered other outcomes as: mortality, other anatomical and organ characteristics, growth evolution, quality of live, other pulmonary endpoints, other cardiac endpoints, sleep apnea, cognitive evaluation, clinical development, visual acuity, and neurological symptoms, long-term adverse events, and death
Type IV-A	Dose and schedule	2 mg/kg/week or every other week of elosulfase alfa	The doses were the same; the study selected was an extension from the randomized trial. However, the placebo arm initiated ERT.
	Age at ERT initiation	5–57 years	5–57 years
	Follow-up time	Follow-up restricted to 24 weeks. No evaluation of long-term outcomes	The follow-up was extended to 120 weeks. Evaluation of long-term outcomes
	Outcomes	6-MWT, a 3-min stair climb test, forced vital capacity, forced expiratory volume in 1 s, health assessment questionnaire, maximum voluntary ventilation, height, growth, earing function, cardiac function, visual acuity, cervical, and lumbar spine examination, biochemical markers, urine keratan sulfate normalization, adverse events, and tolerability	The outcomes evaluated were the same with an extended follow-up
Type VI	Dose and schedule	Weekly infusions of either high (1.0 mg/kg) or low (0.2 mg/kg) doses of galsulfase	It was not clearly specified in most studies. A 1.0 mg/kg weekly infusion dose is assumed
	Age at ERT initiation	Between 5 and 29 years	Between 6 weeks and 59 years
	Follow-up time	The median and maximum duration of follow-up was 11 months. No evaluation of long-term outcomes	The median duration of follow-up was 51 months (range: 6–120 months). No evaluation of long-term outcomes
	Outcomes	12 and 6-MWT, a 3-min stair climb test, urinary glycosaminoglycan levels, joint pain, joint stiffness, physical energy level, assessment of joint range motion, hand dexterity as evidenced by number of coins picked up in 1 min, adverse events, tolerability, and pharmacokinetics	They also considered other outcomes as: mortality, cardiac function, lung function, visual acuity, sleep apnea, auditory function, quality of life, cognitive development, and growth

MPS, Muchopolysaccharidosis; ERT, Enzyme replacement therapy; HSCT, hematopoietic stem cell transplant; 6-MWT, 6-min walk test; IV, Intravenous.

#### Methods Used to Combine Data From Different Study Designs in Each Systematic Review Selected

There were four types of strategies used to combine results from studies of different designs in systematic reviews (see Table 3):

1) The first method was to select studies with the same design and to exclude the other designs (only randomized trials or only prospective nonrandomized studies). The studies excluded are considered with a low level of evidence or inappropriate to cover the research question (El Dib and Pastores, 2007; El Dib, 2009; Brunelli et al., 2016; da Silva et al., 2016; Jameson et al., 2019).
2) The second approximation was to summarize the results of the studies with different designs and discuss the results qualitatively (Alegra et al., 2013; Pal et al., 2015; Schrover et al., 2017; Gomes et al., 2019).
3) The third method was to combine the results of studies with different designs qualitatively, based on a method to assess the strength of the evidence (very low, low, moderate, and high) for each outcome. This approach allows to use a reproducible methodology and to rank each outcome in accordance with its strength of evidence (Bradley et al., 2017; Dornelles et al., 2017; Pérez-López et al., 2017; Perez-Lopez et al., 2018).
4) The fourth method was to define two improvement criteria. One for individual patients included in the meta-analyses (e.g., increase in the 6-min walk test [6-MWT] over the baseline assessment) and another for the specific outcome or group of patients evaluated (e.g., a significant difference versus a 5% null hypothesis in the rate of patients with improvement in the walk test) (Almeida et al., 2018; Sampayo-Cordero et al., 2018; Kuiper et al., 2019; Sampayo-Cordero et al., 2019).

**TABLE 3:** Methods used to combine data from different study designs in each systematic review selected.

Type	Study	Methods used to combine data from different study designs
Types I, II, III, IV, and VI	Pal et al. (2015)	They included different types of study designs and mucopolysaccharidosis. They combine in each mucopolysaccharidosis the results of the same outcomes and types of designs in a meta-analysis. The noncombined results were summarized and discussed
Type I	El Dib and Pastores (2007)	They selected one randomized study. They did not combine results from different study designs
	Dornelles et al. (2017)	1) They combined the median change to baseline or the incidence of some events. Comparison between experimental and control arms were not considered
		2) Qualitative analyses included all the study designs selected. They used GRADE criteria (Guyatt et al. (2008), (2011); Morgan et al. (2019)) to evaluate the strength of evidence for each outcome
	Pérez-López et al.	They used GRADE criteria (Guyatt et al. (2008), (2011); Morgan et al. (2019)) to evaluate the strength of evidence for each outcome
	Sampayo-Cordero et al. (2018a)	They used the percent of clinical cases showing improvement in efficacy outcomes, or no harm in safety outcomes after ERT initiation as a summary of the strength of evidence. A restrictive procedure to aggregate case reports, by selecting standardized and well-defined outcomes, was proposed
	Jameson et al. (2019)	They selected one randomized study. They did not combine results from different study designs
	Kuiper et al. (2019)	1) They graded individual patient data as normal (N) or abnormal (A), both at baseline and at follow-up (based on reference values as reported in the articles). Patients with an abnormal baseline and follow-up (AA) were further classified as stable, improved, or deteriorated at follow-up (AAs, AAi, and AAd)
		2) They graded patient groups as improved, stable, or deteriorated based on quantitative criteria predefined in the original articles
Type II	Alegra et al. (2013)	They used GRADE criteria (Guyatt et al. (2008), (2011); Morgan et al. (2019)) to evaluate the strength of evidence for each outcome
	da Silva et al. (2016)	They selected one randomized study. They did not combine results from different study designs
	Almeida et al. (2018)	They counted the number of infusion reactions, adverse events (serious and nonserious), and deaths in each study
	Pérez-López et al.	They used GRADE criteria (Guyatt et al. (2008), (2011); Morgan et al. (2019)) to evaluate the strength of evidence for each outcome
	Bradley et al. (2017)	They assessed the quality and strength of evidence for each outcome
	Sampayo-Cordero et al. (2019)	They used the percent of clinical cases showing improvement in efficacy outcomes, or no harm in safety outcomes after ERT initiation as a summary of the strength of evidence. A restrictive procedure to aggregate case reports, by selecting standardized and well-defined outcomes, was proposed
Type IV-A	Schrover et al. (2017)	They selected two studies, the phase III clinical trial and their extension. They summarized the results from these studies without combining the results
		Additionally, the authors calculated the minimum clinically important difference in 6-MWT. They calculated it combining the results in a variety of diseases
Type VI	El Dib, (2009)	They combined results from 2 randomized clinical trials. They did not select other study designs
	Brunelli et al. (2016)	They selected one randomized study. They did not combine results from different study designs
	Gomes et al. (2019)	The effectiveness of ERT was identified by a qualitative assessment of each study report. The outcomes were classified as primary and substitutive according to the authors’ assessment

MPS, muchopolysaccharidosis; ERT, enzyme replacement therapy; GRADE, grading of recommendations assessment, development and evaluation; HSCT, hematopoietic stem cell transplant; 6-MWT, 6-min walk test.

#### Rate of Patients Excluded From Systematic Reviews if Case Reports Were Excluded

The rate of patients missing from systematic reviews when case reports were excluded was neither statistically higher than 40% nor higher than 10%. However, the rate of patients tended to be statistically higher than 10% in two studies. Importantly, the systematic reviews with the objective to assess ERT outcomes in the overall population showed the lowest rate of patients excluded (around 3.3%) (Bradley et al., 2017; Gomes et al., 2019; Sampayo-Cordero et al., 2019). Alternatively, the systematic reviews with the objective targeting a specific patient subgroup (ERT initiation in adult age) showed the highest rate of patients excluded (around 16%) (Pérez-López et al., 2017; Pérez-López et al., 2018; Sampayo-Cordero et al., 2018). So, the exclusion of clinical reports is more likely to introduce a bias in subgroup analyses than in the results of the whole population (where the number of assessable patients is higher than those in subgroup analyses) (see Table 4).

**TABLE 4:** The rate of patients excluded from systematic reviews if case reports were excluded.

Type of MPS	Studies with the same target population	Target population	Included in systematic reviews			Rate of patients excluded (95% CI)	*p*-value H0: 10%/40%
			Without case reports	With case reports	Total		
Type I	Pérez-López et al. , Sampayo-Cordero et al. (2018a)	Adult patients	57^a^	11	68	16.2% (8.4–27.1%)	0.074/1
Type II	Bradley et al. (2017), Sampayo-Cordero et al. (2019)	Overall population	928^a^	33	961	3.4% (2.4–4.8%)	1/1
Type II	Pérez-López et al.	Patients > 16 years	3^a^	7	37	18.9% (8–35.2%)	0.072/1
Type VI	Gomes et al. (2019)	Overall population	350	12	362	3.3 (1.7–5.7%)	1/1

CI, confidence interval; H0, null hypothesis.

a The studies included a randomized phase III trial and its follow-up extension. The placebo arm started to receive enzyme replacement therapy. So, the study switched to a prospective nonrandomized design for the long-term outcomes. As the study designs and evaluations performed were different, the information provided for the same patient was different. So, these patients have been considered twice, as participants of the randomized studies and as participants of the nonrandomized studies.

## Discussion

Low clinical trial accrual of patients with rare diseases is an important constraint in evidence-based clinical practice. Additionally, the phenotypic and genotypic heterogeneity also produces a greater fragmentation of the disease and increases the difficulty to generalize the results from only one study (Bradley et al., 2017; Dornelles et al., 2017; Rath et al., 2017). Therefore, the systematic reviews on rare diseases represent a very useful tool for medical community to obtain maximum information from published data and identify areas for improvement (Pérez-López et al., 2017). Over the last years, our comprehension of complex, heterogeneous, highly prevalent diseases has been considerably increased due to significant improvements in molecular and genetic medicine. On the other hand, personalized medicine has fragmented complex diseases into multiple molecular subtypes, each one representing a rare disease (Bartlett and Parelukar, 2017; Jardim et al., 2017; Klein and Gahl, 2018). Thus, the research methods derived from rare diseases and the strategies to integrate multiple heterogeneous data from the literature are highly relevant (Schork, 2015; Pérez-López et al., 2017; Klein and Gahl, 2018; Sampayo-Cordero et al., 2019).

One of the most important items when conducting a systematic review is the assessment of the validity of studies included according to stringent criteria. Usually reviews emphasize the risk of bias in their results. A common practice is to exclude nonrandomized and nonblinded designs (Higgins et al., 2019). However, our results suggest that more than 50% of available patients in the field of MPS are recruited in nonrandomized studies. Methodologies managing missing data suggested that this rate of missing values (>40%) in a usual clinical trial enables results to be used only as hypothesis generators (Little et al., 2012; Madley-Dowd et al., 2019). It would be considered that missingness in a variable collected in a clinical trial is a different concept to the exclusion of patients from nonrandomized studies for systematic reviews. But randomized clinical trials usually provide the strongest methodology to evaluate the efficacy of an intervention (Concato et al., 2000; Higgins et al., 2019). So, it is expected that missingness rates higher than 40% also could bias the results of a systematic review. However, we also considered that these results alone cannot be indicative of bias. Methods for missing data management have stated the relevance of the source of missing data (Little et al., 2012; Madley-Dowd et al., 2019). So, we also observed differences in the baseline characteristics of patients, the conduct of the study, and the outcomes evaluated between randomized and nonrandomized studies. The randomized trials usually recruit pediatric patients (Wraith et al., 2004; Muenzer et al., 2006). Importantly, the clinical manifestations of MPS in adulthood are different from pediatric patients (Lampe et al., 2019b; Stepien et al., 2020). So, the exclusion of nonrandomized studies prevents the assessment of the efficacy and safety of ERT in adulthood. In accordance, previous studies stated that the selection criteria and monitoring conditions of clinical trials usually differed from clinical practice. On the contrary, prospective studies and case reports are the nearest to standard clinical practice conditions (Kahan et al., 2015; Hong et al., 2018; on behalf of the ACMG Professional Practice and Guidelines Committee et al., 2020). We would like to say that excluding nonrandomized studies from a systematic review leads to exclude >50% of available patients and most of the data reported about adult population, alternative treatment schedules, mortality rates, long-term efficacy, and long-term safety.

In addition, some sections of the Cochrane guidelines recommend including only studies with high evidence that “can estimate causality with minimal risk of bias except to examine the case for performing a randomized trial by describing the weakness of the available evidence” (Higgins et al., 2019). In the same direction, some methodologists did not defend that excluding nonrandomized studies from a systematic review results in a selection bias. They argue that “a biased effect estimate from a systematic review may be more harmful to future patients than no estimate at all, particularly if the people using the evidence to make decisions are unaware of its limitations” (Peto et al., 1995; Higgins et al., 2019). However, this recommendation has three important false assumptions.

First, it is assumed that a clinical trial of any research issue is possible (e.g., the assessment of the long-term effects and mortality of an intervention that has improved the disease evolution cannot be carried out using a placebo control. Therefore, no new clinical trials would be performed regardless of how much emphasis is placed on successive reviews (Higgins et al., 2019)).

Second, it is assumed that the lack of epidemiological data is not harmful. However, without published evidence, physicians would be guided by their intuition and their clinical experience acquired from treating a limited number of patients. So, trials with a large sample size will always have a lower risk of bias than trials with a limited number of subjects (Guyatt et al., 2008; Guyatt et al., 2011; Morgan et al., 2019).

Finally, this recommendation assumes that investigators should only be informed of facts where causality can be clearly established. The clinician deals with the patient and relies to a large extent on case reports, especially in rare and ultrarare diseases. Accordingly, there is an established tendency to report the available information with its degree of evidence, rather than not reporting it. The role of nonrandomized data in medical counseling cannot be underestimated (Scarpa et al., 2011; Morgan et al., 2019; Cardoso et al., 2020).

Overall, we consider that excluding most of the data reported about adult population, alternative treatment schedules, mortality rates, long-term efficacy, and long-term safety of a chronic treatment would produce a selection bias that could be easily quantified. In detail, if we excluded findings of nonrandomized studies and “low-quality” trials, a unique clinical trial published in 2004 could be considered. This means ignoring the clinical research done on this disease in the last 16 years (Jameson et al., 2019).

The HSCT represents another form of ERT. The HSCT has been accepted as MPS I therapy for its severe form in the early course of the disease. However, there is not a single RCT on HSCT in the field of MPS. Nonrandomized data have served to establish a consensus on the best target population and its most appropriate strategy of treatment of MPS-I patients (Scarpa et al., 2011). The evidence for these recommendations was not the same weight as if they were based on randomized clinical trials. However, these recommendations have a higher degree of evidence than the individual beliefs of a single investigator; and they had a great utility summarizing the available knowledge to guide clinical practice and the future investigations (Scarpa et al., 2011; Barth and Horovitz, 2018). Nowadays, HSCT is reconsidered as therapy in other forms of neuronopathic MPS after the development of new treatment techniques and the creation of umbilical cords bank and bone marrow donor registries (Barth and Horovitz, 2018).

Accordingly, the Cochrane guidelines accept the inclusion of nonrandomized study designs in systematic reviews under some constraints: “i) when randomized trials are unable to address the effects of the intervention on harm and long-term outcomes or in specific populations or settings; or ii) for interventions that cannot be randomized (e.g., policy change introduced in a single or small number of jurisdictions)” (Higgins et al., 2019).

In addition, the Cochrane guidelines recognize that there is not a general strategy for deciding which nonrandomized studies will be included in a systematic review (section 24.2.1.3). As a possible strategy the authors suggest including either nonrandomized trials with best available designs or nonrandomized studies with a strong design. As the authors suggest, and according to our experience of investigating the long-term outcomes in MPS, the latter strategy may lead to exclude all available studies. So, they recommend giving greater emphasis to the choice of the included studies (Higgins et al., 2019).

These results highlight the relevance of rare disease registries as valuable sources of information for systematic reviews (Montaño et al., 2007; Beck et al., 2014; Wood et al., 2014; Muenzer et al., 2017a, 2017b; Lampe et al., 2019a). Although the methodology presents a higher risk of bias than clinical trials, a global registry is the best alternative for evaluating the long-term efficacy of ERTs and the survival of patients treated with these therapies (Burton et al., 2017). In the absence of data from other designs, it is reasonable to consider these data in the systematic reviews of rare diseases, with an assessment of the methodological flags and possible bias (Bradley et al., 2017; Wikman-Jorgensen et al., 2020).

Usually, case reports are never included in systematic reviews, unless the study indication is an ultrarare disease with only case reports available (Sampayo-Cordero et al., 2018; Sampayo-Cordero et al., 2019). However, our results suggest that the smaller the number of patients in the reference population, the larger the selection bias associated to excluding case reports (Pérez-López et al., 2018; Sampayo-Cordero et al., 2018; Sampayo-Cordero et al., 2019). Accordingly, the selection of case reports should be considered in an MPS systematic review if subgroup analyses were planned (Pérez-López et al., 2017; Pérez-López et al., 2018; Sampayo-Cordero et al., 2018). In addition, we observed that some relevant information about treatment management was usually reported in case reports (Lampe et al., 2019b; Stepien et al., 2020), and previous publications has stated the relevance of case reports to propose clinical novelties (Nakamura et al., 2014; Sampayo-Cordero et al., 2020a).

Alternatively, including nonrandomized studies and case reports raise important questions. The nonrandomized studies have been commonly associated with biased effect estimates, low scientific evidence, and low quality (Morgan et al., 2019; Higgins et al., 2019). Additionally, the data and methods of analyses from randomized and nonrandomized studies are quite different making it difficult to combine both results (Higgins et al., 2019). However, previous studies have stated that properly conducted observational studies and aggregations of case reports could achieve equivalent conclusions than randomized clinical trials and meta-analyses of prospective studies (Concato et al., 2000; Frieden, 2017; Sampayo-Cordero et al., 2018; Sampayo-Cordero et al., 2019). Methods to grade the strength of evidence allow incorporating the results of nonrandomized studies into the data analysis and discussion (Guyatt et al., 2008; Guyatt et al., 2011; Morgan et al., 2019). Accordingly, we observed that the combination of results from different study designs is feasible, communicating the strength of the evidence and the possibility of bias of each recommendation (Dornelles et al., 2017; Kuiper et al., 2019). This is the only way to contrast the short-term efficacy and safety results of clinical trials with the long-term outcomes (Bradley et al., 2017), mortality rates (Burton et al., 2017), alternative treatment schedules (Pérez-López et al., 2017; Sampayo-Cordero et al., 2019), or patient with uncommon phenotype or clinical characteristics of other study types (Pérez-López et al., 2017; Pérez-López et al., 2018).

Our findings described four strategies to combine results from different study designs in systematic reviews. The first strategy was excluding studies with different designs (El Dib and Pastores, 2007; El Dib, 2009; Brunelli et al., 2016; da Silva et al., 2016; Jameson et al., 2019); the second was to summarize the results and discuss them qualitatively (Alegra et al., 2013; Pal et al., 2015; Schrover et al., 2017; Gomes et al., 2019); the third was to combine the results of studies based on a method which assess the strength of the evidence (very low, low, moderate, and high) for each outcome (GRADE) (Bradley et al., 2017; Dornelles et al., 2017; Pérez-López et al., 2017; Perez-Lopez et al., 2018); and the last was to rank individual patients in each study with an improvement or deterioration criterion and combining them quantitatively (Almeida et al., 2018; Sampayo-Cordero et al., 2018; Kuiper et al., 2019; Sampayo-Cordero et al., 2019). Importantly, previous reviews have stated that ranking and aggregation of this patients’ results should be guided by the quality assessment of studies selected and the standardization and good definition of outputs evaluated (Sampayo-Cordero et al., 2018; Sampayo-Cordero et al., 2019).

The second and third strategies are based on interpreting qualitatively the results from different study designs. However, there is an important difference between both methods. The second method proposes to summarize all studies results and discus them without a predefined criterion to combine them. Thus, the interpretation of these results is equivalent to a conventional literature review, although the bibliographic search could be reproducible and more reliable (second strategy) (Alegra et al., 2013; Pal et al., 2015; Schrover et al., 2017; Gomes et al., 2019). The third method proposes to evaluate the strength of evidence for each relevant outcome based on a predefined criterion stated in the protocol and widely accepted. Usually, the results are reported as a qualitative score for each outcome (invaluable, very low, low, moderate, and high evidence grade) (Guyatt et al., 2011; Morgan et al., 2019). So, the interpretation of the results and conclusions are more reproducible and evidence-based. This method could be considered a qualitative meta-analysis (third strategy) (Bradley et al., 2017; Dornelles et al., 2017; Pérez-López et al., 2017; Pérez-López et al., 2018). In accordance with our results, the first strategy can lead to biased and poorly generalizable results in the context of rare diseases. Therefore, results could only be considered as hypothesis generators (exploratory). The second strategy conducts the results without a preplanned strategy, so conclusions should also be considered as exploratory. The third and fourth strategies conduct the analyses with a preplanned and reproducible method. Therefore, we considered them as more reliable source to draw conclusions about the data. It is important to consider that all four methods presented in this review assumed a quality review of the studies selected (Dornelles et al., 2017; Pérez-López et al., 2017; Jameson et al., 2019; Sampayo-Cordero et al., 2019). The first method will use this revision to exclude nonrandomized studies and low-quality clinical trials (Jameson et al., 2019). The second method will comment differences between studies and sources of bias in results or discussion (Dornelles et al., 2017). The third and fourth method will report this quality evaluation in the results section (Pérez-López et al., 2017; Pérez-López et al., 2018; Sampayo-Cordero et al., 2018; Sampayo-Cordero et al., 2019). Thus, these combination methods do not propose to pool results from randomized and nonrandomized trials without carefully evaluating the study quality, with awareness regarding the potential bias in efficacy estimates. On the contrary, these methods propose to evaluate both the quality and heterogeneity of the data. In the third and fourth method authors proposed to report separate estimates from randomized and nonrandomized studies in order to assess if the results could be combined (Morgan et al., 2019; Higgins et al., 2019).

Additionally, conclusions from these studies could not be considered confirmatory without any criticism. The primary source of the data, the proper conduct of the systematic review, and the specific research context should be considered (Higgins et al., 2019).

As it is the case with nonrandomized studies, methods to assess the quality of case reports, to aggregate their results, to analyze the heterogeneity, and to assess publication bias have been also described (Sampayo-Cordero et al., 2018; Sampayo-Cordero et al., 2019). Guidelines to homogenize and upgrade the quality of the case reports are also published (Gagnier et al., 2013), and methods to develop a retrieval system in which, given one case report, one can find other case reports that report the same or very similar main findings have been proposed (Luo et al., 2020).

To analyze the rate of patients excluded, if nonrandomized studies were not included, we have taken advantage of previous systematic reviews to select the individual clinical studies analyzed in this communication. We consider that this strategy provides two important quality control measures. First, the searches and study selection are less affected by the authors’ assumptions. Second, we included communications whose quality is high enough to be selected in previous published systematic reviews. However, an important limitation is that new clinical studies, which were not included in these systematic reviews, are not considered. It is important to state that the most recent publication selected was from 2019 (Gomes et al., 2019; Sampayo-Cordero et al., 2019) and the oldest was from 2007 (El Dib, 2009). In addition, a very recent article in press about a systematic review and meta-analysis, aiming at the evaluation of ERT for treatment of Hunter disease, reported equivalent results that we have reported in this study. Most of the studies selected were nonrandomized, and some important outcomes such as mortality were only analyzed in prospective observational studies (Wikman-Jorgensen et al., 2020).

An important point in our strategy for data analyses is that the patients analyzed in randomized clinical trials and included in nonrandomized follow-up extensions have been considered twice, as participants of randomized and nonrandomized trials (Wraith et al., 2004; Muenzer et al., 2006; Muenzer et al., 2011; Clarke et al., 2009; STRIVE; Investigators et al., 2014; Hendriksz et al., 2016). This strategy makes sense because we are not evaluating the clinical response of each patient. We evaluated the amount of information provided by each design. The fact that they are the same patients is irrelevant since the design of the studies, the outcomes evaluated, and the information provided was different.

It is usually considered that a MEDLINE search alone is not adequate for answering relevant questions (Porter et al., 2020; Higgins et al., 2019). Accordingly, we have increased the scope of our search with the Cochrane and PROSPERO databases, since they are the preferred databases to publish systematic review protocols. The publication of the protocol prior to the analysis is a key recommendation from PRISMA (Stroup et al., 2000; Liberati et al., 2009). This strategy has allowed us to find relevant congress communications (Almeida et al., 2018). Additionally, searches in the LILACS database are increasingly common in systemic review searches (Membrive-Jiménez et al., 2020; Suleiman-Martos et al., 2020). The LILACS database has previously demonstrated its utility incorporating in systematic review’s unique contents, since most of its indexed journals are not indexed in other databases (Clark and Castro, 2001; Clark and Castro, 2002). Accordingly, we found in our study additional systematic reviews in LILACS that were not indexed in other databases (Pal et al., 2015). This was also the case for previous studies performed on MPS and other indications (Pérez-López et al., 2017; Sampayo-Cordero et al., 2018; Sampayo-Cordero et al., 2019; Mösges et al., 2019).

Our study only included systematic reviews from MPS patients. It is important to note that differences between randomized and nonrandomized studies observed in our analyses have also been described in other highly prevalent and rare diseases. In addition, the research in ultrarare genetic diseases is usually based on registry studies, analysis of public databases, and communication of case reports. Some ultrarare diseases have a proportion of nonrandomized studies higher than those in our study. (Nakamura et al., 2014; Kahan et al., 2015; Hong et al., 2018; Jansen-van der Weide et al., 2018; on behalf of the ACMG Professional Practice and Guidelines Committee et al., 2020). Therefore, the recommendations obtained from this meta-analysis could be extended to other rare diseases.

## Conclusion

More than 50% of patients analyzed in MPS publications have been recruited in nonrandomized studies (Bradley et al., 2017; Dornelles et al., 2017). The baseline characteristics of the recruited patients, the duration of follow-up, and the clinical outcomes evaluated differed between the randomized and nonrandomized studies (El Dib, 2009; Brunelli et al., 2016; Gomes et al., 2019). Therefore, our findings recommend including nonrandomized studies in the systematic reviews of MPS to increase the representativeness of the results and avoid a selection bias. Despite the difficulties in analyzing the results of different designs all together, previous studies have proposed multiple methods to combine them (Bradley et al., 2017; Pérez-López et al., 2017; Sampayo-Cordero et al., 2018; Kuiper et al., 2019). Additionally, results suggest the relevance of including case reports in a systematic review, since smaller the number of patients in the reference population, larger the selection bias associated to excluding case reports. Therefore, the selection of case reports should be considered in an MPS systematic review if subgroup analyses were to be planned (Pérez-López et al., 2018; Sampayo-Cordero et al., 2018; Sampayo-Cordero et al., 2019). In addition, differences between randomized and nonrandomized studies observed in our analyses are observed in other diseases as well (Concato et al., 2000; Liberati et al., 2009; Frieden, 2017; Hong et al., 2018). Therefore, the recommendations obtained from this study should be considered when conducting systematic reviews on rare diseases.

## Data Availability

All data generated or analyzed during this study are included in this published article and its information files.

## References

[B1] Alegra T. Eizerik D. P. Cerqueira C. C. S. d. Pereira T. V. Dornelles A. D. Schwartz I. V. D. (2013). Eficácia e segurança da terapia com idursulfase em pacientes com mucopolissacaridose tipo II, com e sem comparação com placebo: revisão sistemática e metanálise. Cad. Saúde Pública 29, s45–s58. 10.1590/0102-311X00017613 25402250

[B2] Almeida P. H. R. F. Lemos L. L. P. D. Alvares J. Godman B. Bennie M. Acurcio F. D. A. (2018). Safety of Enzyme Replacement Therapy for Mucopolysaccharidosis II. Ital J Pediatr. 44(Suppl. 2): 120, 10.13140/RG.2.2.31328.74242

[B3] Barth A. L. Horovitz D. D. G. (2018). Hematopoietic Stem Cell Transplantation in Mucopolysaccharidosis Type II. J. Inborn Errors Metab. Screen. 6, 232640981877909. 10.1177/2326409818779097

[B4] Bartlett J. M. S. Parelukar W. (2017). Breast Cancers Are Rare Diseases-And Must Be Treated as Such. npj Breast Cancer 3, 11. 10.1038/s41523-017-0013-y 28649651 PMC5460259

[B5] Beck M. Arn P. Giugliani R. Muenzer J. Okuyama T. Taylor J. (2014). The Natural History of MPS I: Global Perspectives From the MPS I Registry. Genet. Med. 16, 759–765. 10.1038/gim.2014.25 24675674 PMC4189384

[B6] Bradley L. A. Haddow H. R. M. Palomaki G. E. (2017). Treatment of Mucopolysaccharidosis Type II (Hunter Syndrome): Results from a Systematic Evidence Review. Genet. Med. 19, 1187–1201. 10.1038/gim.2017.30 28640238

[B7] Brunelli M. J. Atallah Á. N. da Silva E. M. (2016). Enzyme Replacement Therapy With Galsulfase for Mucopolysaccharidosis Type VI. Cochrane Database Syst. Rev. 3, CD009806. 10.1002/14651858.CD009806.pub2 26943923

[B8] Burton B. K. Jego V. Mikl J. Jones S. A. (2017). Survival in Idursulfase-Treated and Untreated Patients with Mucopolysaccharidosis Type II: Data from the Hunter Outcome Survey (HOS). J. Inherit. Metab. Dis. 40, 867–874. 10.1007/s10545-017-0075-x 28887757

[B9] Cardoso F. Paluch-Shimon S. Senkus E. Curigliano G. Aapro M. S. André F. (2020). 5th ESO-ESMO International Consensus Guidelines for Advanced Breast Cancer (ABC 5). Ann. Oncol. 31, 1623–1649. 10.1016/j.annonc.2020.09.010 32979513 PMC7510449

[B10] Catalá-López F. Hutton B. Driver J. A. Page M. J. Ridao M. Valderas J. M. (2017). Cancer and central Nervous System Disorders: Protocol for an Umbrella Review of Systematic Reviews and Updated Meta-Analyses of Observational Studies. Syst. Rev. 6, 69. 10.1186/s13643-017-0466-y 28376926 PMC5379758

[B11] Clark O. A. Castro A. A. (2001). Cochrane Reviews Must Use LILACS Database Like Source of Articles. BMC News and views 1. 10.1186/2048-4623-1-S3-PB081

[B12] Clark O. A. C. Castro A. A. (2002). Searching the Literatura Latino Americana e Do Caribe em Ciências da Saúde (LILACS) Database Improves Systematic Reviews. Int. J. Epidemiol. 31, 112–114. 10.1093/ije/31.1.112 11914305

[B13] Clarke L. A. Wraith J. E. Beck M. Kolodny E. H. Pastores G. M. Muenzer J. (2009). Long-Term Efficacy and Safety of Laronidase in the Treatment of Mucopolysaccharidosis I. PEDIATRICS 123, 229–240. 10.1542/peds.2007-3847 19117887

[B14] Concato J. Shah N. Horwitz R. I. (2000). Randomized, Controlled Trials, Observational Studies, and the Hierarchy of Research Designs. N. Engl. J. Med. 342, 1887–1892. 10.1056/NEJM200006223422507 10861325 PMC1557642

[B15] da Silva E. M. Strufaldi M. W. L. Andriolo R. B. Silva L. A. (2016). Enzyme Replacement Therapy With Idursulfase for Mucopolysaccharidosis Type II (Hunter Syndrome). Cochrane Database Syst. Rev. 2 (2), CD008185. 10.1002/14651858.CD008185.pub4 26845288 PMC7173756

[B16] Dornelles A. D. Artigalás O. da Silva A. A. Ardila D. L. V. Alegra T. Pereira T. V. (2017). Efficacy and Safety of Intravenous Laronidase for Mucopolysaccharidosis Type I: A Systematic Review and Meta-Analysis. PLOS ONE 12, e0184065. 10.1371/journal.pone.0184065 28859139 PMC5578671

[B17] El Dib R. (2009). A Systematic Review of New Advances in the Management of Mucopolysaccharidosis VI (Maroteaux-Lamy Syndrome): Focus on Galsulfase. Biologics: Targets Ther. 3:459–468. 10.2147/BTT.2009.3580 PMC2763316 19851471

[B18] El Dib R. P. Pastores G. M. (2007). Laronidase for Treating Mucopolysaccharidosis Type I. Genet. Mol. Res. 6, 667–674. 18050087

[B19] Frieden T. R. (2017). Evidence for Health Decision Making - Beyond Randomized, Controlled Trials. N. Engl. J. Med. 377, 465–475. 10.1056/NEJMra1614394 28767357

[B20] Gagnier J. J. Kienle G. Altman D. G. Moher D. Sox H. Riley D. (2013). The CARE Guidelines: Consensus-Based Clinical Case Reporting Guideline Development. Case Rep. 2013, bcr2013201554. 10.1136/bcr-2013-201554 PMC3844611 24228906

[B21] Gomes D. F. Gallo L. G. Leite B. F. Silva R. B. da Silva E. N. (2019). Clinical Effectiveness of Enzyme Replacement Therapy with Galsulfase in Mucopolysaccharidosis Type VI Treatment: Systematic Review. Jrnl Inher Metab. Disea 42, 66–76. 10.1002/jimd.12028 30740728

[B22] Guyatt G. H. Oxman A. D. Vist G. E. Kunz R. Falck-Ytter Y. Alonso-Coello P. (2008). GRADE: an Emerging Consensus on Rating Quality of Evidence and Strength of Recommendations. BMJ 336, 924–926. 10.1136/bmj.39489.470347.AD 18436948 PMC2335261

[B23] Guyatt G. Oxman A. D. Akl E. A. Kunz R. Vist G. Brozek J. (2011). GRADE Guidelines: 1. Introduction-GRADE Evidence Profiles and Summary of Findings Tables. J. Clin. Epidemiol. 64, 383–394. 10.1016/j.jclinepi.2010.04.026 21195583

[B24] Hendriksz C. J. Burton B. Fleming T. R. Harmatz P. Hughes D. Jones S. A. (2014). Efficacy and Safety of Enzyme Replacement Therapy With BMN 110 (Elosulfase Alfa) for Morquio A Syndrome (Mucopolysaccharidosis IVA): a Phase 3 Randomised Placebo-Controlled Study. J. Inherit. Metab. Dis. 37, 979–990. 10.1007/s10545-014-9715-6 24810369 PMC4206772

[B25] Hendriksz C. J. Parini R. AlSayed M. D. Raiman J. Giugliani R. Solano Villarreal M. L. (2016). Long-term Endurance and Safety of Elosulfase Alfa Enzyme Replacement Therapy in Patients With Morquio A Syndrome. Mol. Genet. Metab. 119, 131–143. 10.1016/j.ymgme.2016.05.018 27380995

[B26] Higgins J. Thomas J. Chandler J. Cumpston M. Li T. Page M. J. (2019). Cochrane handbook for systematic reviews of interventions. (Accessed June 8, 2020).

[B27] Hong J.-L. Jonsson Funk M. LoCasale R. Dempster S. E. Cole S. R. Webster-Clark M. (2018). Generalizing Randomized Clinical Trial Results: Implementation and Challenges Related to Missing Data in the Target Population. Am. J. Epidemiol. 187, 817–827. 10.1093/aje/kwx287 29020193 PMC5888945

[B28] Jameson E. Jones S. Remmington T. (2019). Enzyme Replacement Therapy with Laronidase (Aldurazyme) for Treating Mucopolysaccharidosis Type I. Cochrane Database Syst. Rev. 6 (6), CD009354. 10.1002/14651858.CD009354.pub5 31211405 PMC6581069

[B29] Jansen-van der Weide M. C. Gaasterland C. M. W. Roes K. C. B. Pontes C. Vives R. Sancho A. (2018). Rare Disease Registries: Potential Applications Towards Impact on Development of New Drug Treatments. Orphanet J. Rare Dis. 13(1):154. 10.1186/s13023-018-0836-0 30185208 PMC6126025

[B30] Jardim D. L. Groves E. S. Breitfeld P. P. Kurzrock R. (2017). Factors Associated with Failure of Oncology Drugs in Late-Stage Clinical Development: A Systematic Review. Cancer Treat. Rev. 52, 12–21. 10.1016/j.ctrv.2016.10.009 27883925

[B31] Kahan B. C. Rehal S. Cro S. (2015). Risk of Selection Bias in Randomised Trials. Trials 16, 405. 10.1186/s13063-015-0920-x 26357929 PMC4566301

[B32] Klein C. Gahl W. A. (2018). Patients with Rare Diseases: From Therapeutic Orphans to Pioneers of Personalized Treatments. EMBO Mol. Med. 10, 1–3. 10.15252/emmm.201708365 29180354 PMC5760852

[B33] Kuiper G. A. Nijmeijer S. C. M. Roelofs M. J. M. Lee J. H. Hollak C. E. M. Bosch A. M. (2019). Limited Data to Evaluate Real‐World Effectiveness of Enzyme Replacement Therapy for Mucopolysaccharidosis Type I. Jrnl Inher Metab. Disea 42, 762–775. 10.1002/jimd.12103 31020996

[B34] Lampe C. Harmatz P. R. Parini R. Sharma R. Teles E. L. Johnson J. (2019a). Enzyme Replacement Therapy Initiated in Adulthood: Findings From the Mucopolysaccharidosis VI Clinical Surveillance Program. Mol. Genet. Metab. 127, 355–360. 10.1016/j.ymgme.2019.06.008 31324526

[B35] Lampe C. McNelly B. Gevorkian A. K. Hendriksz C. J. Lobzhanidze T. V. Pérez-López J. (2019b). Transition of Patients With Mucopolysaccharidosis From Paediatric to Adult Care. Mol. Genet. Metab. Rep. 21, 100508. 10.1016/j.ymgmr.2019.100508 31687335 PMC6819742

[B36] Liberati A. Altman D. G. Tetzlaff J. Mulrow C. Gotzsche P. C. Ioannidis J. P. A. (2009). The PRISMA Statement for Reporting Systematic Reviews and Meta-Analyses of Studies That Evaluate Healthcare Interventions: Explanation and Elaboration. BMJ 339, b2700. 10.1136/bmj.b2700 19622552 PMC2714672

[B37] Little R. J. D'Agostino R. Cohen M. L. Dickersin K. Emerson S. S. Farrar J. T. (2012). The Prevention and Treatment of Missing Data in Clinical Trials. N. Engl. J. Med. 367, 1355–1360. 10.1056/NEJMsr1203730 23034025 PMC3771340

[B38] Luo M. Cohen A. M. Addepalli S. Smalheiser N. R. (2020). Identifying Main Finding Sentences in Clinical Case Reports, Database, 2020, baaa041. 10.1093/database/baaa041 32525207 PMC7287507

[B39] Madley-Dowd P. Hughes R. Tilling K. Heron J. (2019). The Proportion of Missing Data Should Not Be Used to Guide Decisions on Multiple Imputation. J. Clin. Epidemiol. 110, 63–73. 10.1016/j.jclinepi.2019.02.016 30878639 PMC6547017

[B40] ACMG Professional Practice and Guidelines Committee Malinowski J. Miller D. T. Miller D. T. Demmer L. Gannon J. Pereira E. M. (2020). Systematic Evidence-Based Review: Outcomes from Exome and Genome Sequencing for Pediatric Patients with Congenital Anomalies or Intellectual Disability. Genet. Med. 22, 986–1004. 10.1038/s41436-020-0771-z 32203227 PMC7222126

[B41] Membrive-Jiménez M. J. Pradas-Hernández L. Suleiman-Martos N. Vargas-Román K. Cañadas-De la Fuente G. A. Gomez-Urquiza J. L. (2020). Burnout in Nursing Managers: A Systematic Review and Meta-Analysis of Related Factors, Levels and Prevalence. Ijerph 17, 3983. 10.3390/ijerph17113983 PMC7312638 32512738

[B42] Montaño A. M. Tomatsu S. Gottesman G. S. Smith M. Orii T. (2007). International Morquio A Registry: Clinical Manifestation and Natural Course of Morquio A Disease. J. Inherit. Metab. Dis. 30, 165–174. 10.1007/s10545-007-0529-7 17347914

[B43] Morgan R. L. Thayer K. A. Santesso N. Holloway A. C. Blain R. Eftim S. E. (2019). A Risk of Bias Instrument for Non-randomized Studies of Exposures: A Users' Guide to its Application in the Context of GRADE. Environ. Int. 122, 168–184. 10.1016/j.envint.2018.11.004 30473382 PMC8221004

[B44] Mösges R. Valero Santiago A. Allekotte S. Jahed N. Astvatsatourov A. Sager A. (2019). Subcutaneous Immunotherapy With Depigmented-Polymerized Allergen Extracts: a Systematic Review and Meta-Analysis. Clin. Transl Allergy 9. 10.1186/s13601-019-0268-5 PMC6549305 31171962

[B45] Muenzer J. Beck M. Eng C. M. Giugliani R. Harmatz P. Martin R. (2011). Long-Term, Open-Labeled Extension Study of Idursulfase in the Treatment of Hunter Syndrome. Genet. Med. 13, 95–101. 10.1097/GIM.0b013e3181fea459 21150784

[B46] Muenzer J. Giugliani R. Scarpa M. Tylki-Szymańska A. Jego V. Beck M. (2017a). Clinical Outcomes in Idursulfase-Treated Patients With Mucopolysaccharidosis Type II: 3-year Data From the Hunter Outcome Survey (HOS). Orphanet J. Rare Dis. 12(1):161. 10.1186/s13023-017-0712-3 28974237 PMC5627440

[B47] Muenzer J. Jones S. A. Tylki-Szymańska A. Harmatz P. Mendelsohn N. J. Guffon N. (2017b). Ten Years of the Hunter Outcome Survey (HOS): Insights, Achievements, and Lessons Learned From a Global Patient Registry. Orphanet J. Rare Dis. 12(1):82. 10.1186/s13023-017-0635-z 28464912 PMC5414331

[B48] Muenzer J. Wraith J. E. Beck M. Giugliani R. Harmatz P. Eng C. M. (2006). A Phase II/III Clinical Study of Enzyme Replacement Therapy With Idursulfase in Mucopolysaccharidosis II (Hunter Syndrome). Genet. Med. 8, 465–473. 10.1097/01.gim.0000232477.37660.fb 16912578

[B49] Nakamura T. Igarashi H. Ito T. Jensen R. T. (2014). Important of Case-Reports/series, in Rare Diseases: Using Neuroendocrine Tumors as an Example. Wjcc 2, 608–613. 10.12998/wjcc.v2.i11.608 25405184 PMC4233424

[B50] Page M. J. Moher D. (2017). Evaluations of the Uptake and Impact of the Preferred Reporting Items for Systematic Reviews and Meta-Analyses (PRISMA) Statement and Extensions: a Scoping Review. Syst. Rev. 6, 263. 10.1186/s13643-017-0663-8 29258593 PMC5738221

[B51] Pal A. R. Brown N. Jones S. A. Bigger B. W. Bruce I. A. (2015). Obstructive Sleep Apnea in MPS. J. Inborn Errors Metab. Screen. 3, 232640981561639. 10.1177/2326409815616392

[B52] Pérez-López J. Moltó-Abad M. Muñoz-Delgado C. Morales-Conejo M. Ceberio-Hualde L. del Toro M. (2018). Efficacy of Idursulfase Therapy in Patients with Mucopolysaccharidosis Type II Who Initiated Enzyme Replacement Therapy in Adult Age. A Systematic Review of the Literature. Mol. Genet. Metab. 124, 216–227. 10.1016/j.ymgme.2018.04.013 29801985

[B53] Pérez-López J. Morales-Conejo M. López-Rodríguez M. Hermida-Ameijeiras Á. Moltó-Abad M. (2017). Efficacy of Laronidase Therapy in Patients with Mucopolysaccharidosis Type I Who Initiated Enzyme Replacement Therapy in Adult Age. A Systematic Review and Meta-Analysis. Mol. Genet. Metab. 121, 138–149. 10.1016/j.ymgme.2017.04.004 28410878

[B54] Peto R. Collins R. Gray R. (1995). Large-scale Randomized Evidence: Large, Simple Trials and Overviews of Trials. J. Clin. Epidemiol. 48, 23–40. 10.1016/0895-4356(94)00150-O 7853045

[B55] Porter L. Shoushtarizadeh A. Jelinek G. A. Brown C. R. Lim C. K. de Livera A. M. (2020). Metabolomic Biomarkers of Multiple Sclerosis: A Systematic Review. Front. Mol. Biosci. 7, 574133. 10.3389/fmolb.2020.574133 33381517 PMC7768024

[B56] Rath A. Salamon V. Peixoto S. Hivert V. Laville M. Segrestin B. (2017). A Systematic Literature Review of Evidence-Based Clinical Practice for Rare Diseases: what Are the Perceived and Real Barriers for Improving the Evidence and How Can They Be Overcome? Trials 18. 10.1186/s13063-017-2287-7 PMC5700662 29166947

[B57] Reeves B. C. Wells G. A. Waddington H. (2017). Quasi-experimental Study Designs Series-Paper 5: a Checklist for Classifying Studies Evaluating the Effects on Health Interventions-A Taxonomy Without Labels. J. Clin. Epidemiol. 89, 30–42. 10.1016/j.jclinepi.2017.02.016 28351692 PMC5669452

[B58] Sampayo-Cordero M. Miguel-Huguet B. Malfettone A. Pérez-García J. M. Llombart-Cussac A. Cortés J. (2020a). The Value of Case Reports in Systematic Reviews from Rare Diseases. The Example of Enzyme Replacement Therapy (ERT) in Patients With Mucopolysaccharidosis Type II (MPS-II). Ijerph 17, 6590. 10.3390/ijerph17186590 PMC7558586 32927819

[B59] Sampayo-Cordero M. Miguel-Huguet B. Pardo-Mateos A. Malfettone A. Pérez-García J. Llombart-Cussac A. (2019). Agreement Between Results of Meta-Analyses From Case Reports and Clinical Studies, Regarding Efficacy and Safety of Idursulfase Therapy in Patients With Mucopolysaccharidosis Type II (MPS-II). A New Tool for Evidence-Based Medicine in Rare Diseases. Orphanet J. Rare Dis. 14, 230. 10.1186/s13023-019-1202-6 31639024 PMC6805333

[B60] Sampayo-Cordero M. Miguel-Huguet B. Pardo-Mateos A. Moltó-Abad M. Muñoz-Delgado C. Pérez-López J. (2018). Agreement between the Results of Meta-Analyses from Case Reports and From Clinical Studies Regarding the Efficacy of Laronidase Therapy in Patients with Mucopolysaccharidosis Type I Who Initiated Enzyme Replacement Therapy in Adult Age: An Example of Case Reports Meta-Analyses as an Useful Tool for Evidence-Based Medicine in Rare Diseases. Mol. Genet. Metab. 123, 69–75. 10.1016/j.ymgme.2018.01.002 29336994

[B61] Sampayo-Cordero M. Miguel-Huguet B. Pérez-García J. Páez D. Guerrero-Zotano Á. L. Garde-Noguera J. (2020b). Inclusion of Non-inferiority Analysis in Superiority-Based Clinical Trials With Single-Arm, Two-Stage Simon’s Design. Contemp. Clin. Trials Commun. 20, 100678. 10.1016/j.conctc.2020.100678 33336109 PMC7733004

[B62] Scarpa M. Almássy Z. Beck M. Bodamer O. Bruce I. A. De Meirleir L. (2011). Mucopolysaccharidosis Type II: European Recommendations for the Diagnosis and Multidisciplinary Management of a Rare Disease. Orphanet J. Rare Dis. 6, 72. 10.1186/1750-1172-6-72 22059643 PMC3223498

[B63] Schork N. J. (2015). Personalized Medicine: Time for One-Person Trials. Nature 520, 609–611. 10.1038/520609a 25925459

[B64] Schrover R. Evans K. Giugliani R. Noble I. Bhattacharya K. (2017). Minimal Clinically Important Difference for the 6-min Walk Test: Literature Review and Application to Morquio A Syndrome. Orphanet J. Rare Dis. 12(1):78. 10.1186/s13023-017-0633-1 28441951 PMC5405472

[B65] Stepien K. M. Gevorkyan A. K. Hendriksz C. J. Lobzhanidze T. V. Pérez-López J. Tol G. (2020). Critical Clinical Situations in Adult Patients With Mucopolysaccharidoses (MPS). Orphanet J. Rare Dis. 15(1):114. 10.1186/s13023-020-01382-z 32410642 PMC7227065

[B66] Stroup D. F. Berlin J. A. Morton S. C. Olkin I. Williamson G. D. Rennie D. (2000). Meta-analysis of Observational Studies in Epidemiology: a Proposal for Reporting. Meta-Analysis of Observational Studies in Epidemiology (MOOSE) Group. JAMA 283(15):2008–2012. 10.1001/jama.283.15.2008 10789670

[B67] Suleiman-Martos N. Albendín-García L. Gómez-Urquiza J. L. Vargas-Román K. Ramirez-Baena L. Ortega-Campos E. (2020). Prevalence and Predictors of Burnout in Midwives: A Systematic Review and Meta-Analysis. Ijerph 17, 641. 10.3390/ijerph17020641 PMC7013833 31963831

[B68] U.S. Department of Health and Human Services Food and Drug Administration (2017). Center for Drug Evaluation and Research (CDER), and Center for Biologics Evaluation and Research (CBER)Multiple Endpoints in Clinical Trials - Guidance for Industry, Available at: https://www.fda.gov/downloads/drugs/guidancecomplianceregulatoryinformation/guidances/ucm536750.pdf

[B69] Wikman-Jorgensen P. E. López Amorós A. Peris García J. Esteve Atienzar P. J. Cañizares Navarro R. Asensio Tomás M. L. (2020). Enzyme Replacement Therapy for the Treatment of Hunter Disease: A Systematic Review With Narrative Synthesis and Meta-Analysis. Mol. Genet. Metab. 131, 206–210. 10.1016/j.ymgme.2020.07.005 32773276

[B70] Wood J. Siedman S. Siedman J. Levy P. Brown K. Wilson S. (2014). Sanfilippo Syndrome Registry Project and Natural History Studies: an Example of Patients, Parents and Researchers Collaborating for a Cure. Mol. Genet. Metab. 111, S115. 10.1016/j.ymgme.2013.12.287

[B71] Wraith J. E. Clarke L. A. Beck M. Kolodny E. H. Pastores G. M. Muenzer J. (2004). Enzyme Replacement Therapy for Mucopolysaccharidosis I: a Randomized, Double-Blinded, Placebo-Controlled, Multinational Study of Recombinant Human α-L-Iduronidase (Laronidase). J. Pediatr. 144, 581–588. 10.1016/j.jpeds.2004.01.046 15126990

